# A machine learning approach for differentiating malignant from benign enhancing foci on breast MRI

**DOI:** 10.1186/s41747-019-0131-4

**Published:** 2020-01-28

**Authors:** Natascha C. D’Amico, Enzo Grossi, Giovanni Valbusa, Francesca Rigiroli, Bernardo Colombo, Massimo Buscema, Deborah Fazzini, Marco Ali, Ala Malasevschi, Gianpaolo Cornalba, Sergio Papa

**Affiliations:** 1 Unit of Diagnostic Imaging and Stereotactic Radiotherapy, Centro Diagnostico Italiano S.p.A., Via Saint Bon 20, 20147 Milan, Italy; 2 Computer Systems & Bioinformatics Laboratory Department of Engineering, University Campus Bio-Medico of Rome, Via Álvaro del Portillo 21, 00128 Rome, Italy; 3 Bracco Imaging S.p.A., Via Egidio Folli 50, 20134 Milan, Italy; 4 Università degli Studi di Milano, Scuola di specializzazione di Radiodiagnostica, Via Festa del Perdono 7, Milan, Italy; 5 Centro Ricerche Semeion, Via Sersale 117, 00128 Rome, Italy

**Keywords:** Artificial intelligence, Breast neoplasms, Gadobenic acid, Machine learning, Magnetic resonance imaging

## Abstract

**Background:**

Differentiate malignant from benign enhancing foci on breast magnetic resonance imaging (MRI) through radiomic signature.

**Methods:**

Forty-five enhancing foci in 45 patients were included in this retrospective study, with needle biopsy or imaging follow-up serving as a reference standard. There were 12 malignant and 33 benign lesions. Eight benign lesions confirmed by over 5-year negative follow-up and 15 malignant histopathologically confirmed lesions were added to the dataset to provide reference cases to the machine learning analysis. All MRI examinations were performed with a 1.5-T scanner. One three-dimensional T1-weighted unenhanced sequence was acquired, followed by four dynamic sequences after intravenous injection of 0.1 mmol/kg of gadobenate dimeglumine. Enhancing foci were segmented by an expert breast radiologist, over 200 radiomic features were extracted, and an evolutionary machine learning method (“training with input selection and testing”) was applied. For each classifier, sensitivity, specificity and accuracy were calculated as point estimates and 95% confidence intervals (CIs).

**Results:**

A *k*-nearest neighbour classifier based on 35 selected features was identified as the best performing machine learning approach. Considering both the 45 enhancing foci and the 23 additional cases, this classifier showed a sensitivity of 27/27 (100%, 95% CI 87–100%), a specificity of 37/41 (90%, 95% CI 77–97%), and an accuracy of 64/68 (94%, 95% CI 86–98%).

**Conclusion:**

This preliminary study showed the feasibility of a radiomic approach for the characterisation of enhancing foci on breast MRI.

## Key points

- Radiomic signature could distinguish malignant from benign enhancing foci on magnetic resonance imaging of the breast
- In this study, we applied a “training with input selection and testing “machine learning algorithm on 45 foci, using 8 confirmed benign lesions and 15 confirmed malignant lesions as reference cases
- Over 200 radiomic features were extracted.
- Overall, a *k*-nearest neighbour classifier based on 35 selected features showed an over 90% accuracy.

## Background

Contrast-enhanced magnetic resonance imaging (MRI) has emerged as a non-invasive radiation-free imaging technique for the detection and diagnosis of breast lesions, substantially influencing the diagnosis, prognosis, and treatment of patients with breast cancer [1–6].

This technique is able to detect also small enhancing lesions, with 5 mm or lower maximum diameter, which might be difficult to further characterise. These small findings were defined by the American College of Radiology Breast Imaging Reporting and Data System (BI-RADS) as enhancing foci [7]. Depending on the spatial resolution, it is difficult to evaluate their morphology and dynamic behaviour, while the small size makes difficult to perform MRI-guided needle biopsy, so that their changes are commonly longitudinally monitored with serial examinations to reach a conclusive diagnosis [8]. Foci were frequently associated with an increased hormonal stimulation, but they can also represent the early onset of a malignant lesion [9, 10]. Studies addressing the malignancy rate of foci showed highly variable results, ranging from 2 to 23% [9–13]. Thus, the best management of foci is still under discussion. The issue is of particular interest in high-risk women, especially considering the importance of early diagnosis in this group of patients.

Until the recent rising of radiomics [14], computer-based medical image analysis was focused on computer-aided detection systems supporting the identification of suspicious lesions deserving the attention of the radiologist and on computer-aided diagnosis systems, assisting radiologists in decision-making [15]. Although radiomics was a natural evolution of these systems, the objectives of the two approaches were different. While computer-aided detection or diagnosis systems aimed at delivering a single answer (*i.e.* presence/absence of lesions; malignant *versus* benign differentiation), radiomics was designed to combine radiomic data from images with patient history, risk factors, clinical investigation, and other patient information to provide more powerful decision support models [16]. Radiomics assumes that medical images contain quantitative information that radiologists are not able to perceive and that may be correlated to clinical end-points (such as lesion nature or evolution as well as predictive information about treatment efficacy) based on big data. Although there were no universally recognised guidelines yet, the radiomic workflow consists usually of the following main steps [16]: clinical data and images collection; image segmentation, features extraction (*i.e.* to obtain quantitative information about the tissue, also called “descriptors”); definition of a machine learning (ML) model and model validation, preferably against an independent dataset.

The aim of this observational retrospective study was to test the ability to differentiate malignant from benign foci on breast MRI through radiomic signature.

## Methods

### Study design and population

The local Ethics Committee of Fondazione IRCCS Ca’ Granda Ospedale Maggiore Policlinico approved this retrospective study (protocol code CE-MRm; approved on December 13, 2018). This study was supported by local research funds of CDI Centro Diagnostico Italiano, a clinical diagnostic centre. Due to the retrospective nature of this study, no specific informed consent was necessary. In this observational retrospective study, we reviewed contrast-enhanced breast MRI examinations performed at our Institution between January 2012 and December 2017, to create training/testing sets on which to apply and evaluate the performance of our algorithms.

This data set consisted of:

- Patients with contrast-enhancing breast foci (enhancing lesions smaller than 5 mm in diameter) with definitive characterisation (benign or malignant) confirmed by histopathology or with breast MRI or ultrasound examination performed after at least 1 year
- Patients with benign breast lesions with 5 years of MRI stability (unambiguous cases)
- Patients with malignant breast lesions histopathologically confirmed (unambiguous cases)

Patients with incomplete or negative breast MRI examinations were excluded. Breast foci were defined following the ACR BI-RADS Atlas® 5th edition as tiny dots of enhancement that does not clearly represent a space-occupying lesion or mass and does not clearly show a mass on unenhanced imaging [17].

### MRI protocol

Images were acquired on a 1.5-T scanner (Philips Achieva, Philips Medical Systems, Best, The Netherlands). According to clinical practice, examinations were performed with the patient laying in prone position, with the breasts inserted into a surface 16-channel phase-array coil. The sequence taken into account was an axial T1-weighted fast field-echo including inversion recovery with spectral attenuated fat suppression, with a repetition time of 5.1 ms, an echo time of 2.5 ms, a slice thickness of 1 mm, and a field of view of 340 × 340 mm (in-plane resolution 1 × 1 mm). The protocol consisted of one unenhanced and four contrast sequences, with a temporal resolution of 60 s. Gadobenate dimeglumine (Multihance®, Bracco, Milan, Italy) was used as a contrast agent at the dose of 0.1 mmol/kg (0.2 mL/kg); the injection rate was 2 mL/s. The images acquired soon after contrast agent injection were compared to the unenhanced ones.

### Image and data analysis

#### Registration

MRI series were slice-wise co-registered to compensate for patient motion. Registration was done using the ImageJ StackReg plugin [18], based on an automatic subpixel registration algorithm that minimises the mean square difference of intensities between a target and a floating image [16]. Briefly, subvolumes of the MRI volume including lesions and surrounding tissues were cropped from unenhanced and contrast-enhanced datasets. After that, the five extracted subvolumes were automatically slice-wise co-registered by two-dimensional-affine transform. The accuracy of co-registration was assessed by an expert radiologist.

#### Segmentation

**Fig. 1:**
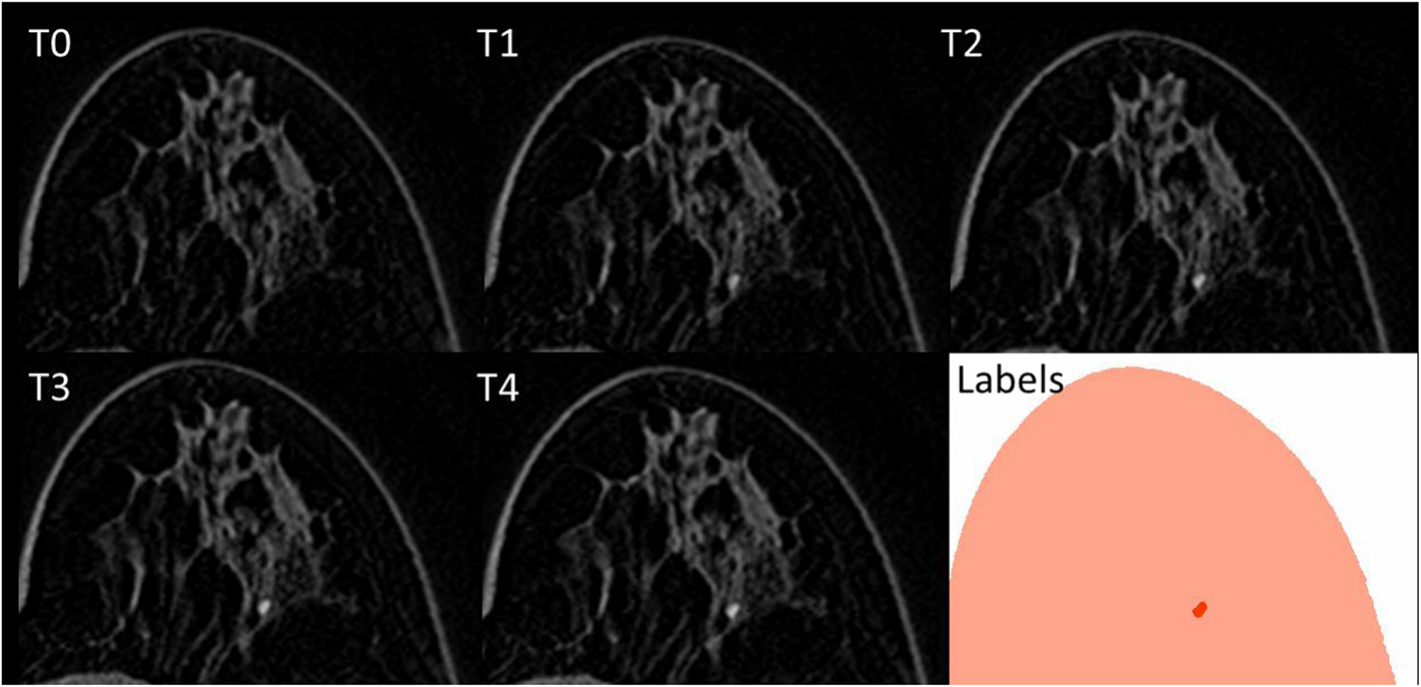
Breast magnetic resonance imaging showing in T0 the first (unenhanced) image and from T1 to T4 the contrast-enhanced images, where the wash-in and wash-out phenomena give information about the malignant or benign nature of the lesion. In the last image (“labels”), the segmented focus is coloured in red while normal breast tissues are coloured in pink

Manual lesion segmentation was carried out by one expert radiologist with more than 10 years of experience in breast MRI on the co-registered images using ImageJ [19]. Due to the spatial coherence of the unenhanced and enhanced images after co-registration, only one lesion mask was defined for each contrast-enhanced series. Images defining segmented lesion areas for each slice were defined as label images. Label and contrast-enhanced images were cropped to the bounding box containing lesions to avoid the analysis of unnecessary parts of the image. In Fig. 1, an example of a focus on unenhanced (T0) and contrast-enhanced (T1–T4) images, with its segmentation, is shown.

#### Feature extraction, selection, and classification

Features were calculated using a dedicated software developed in C++ based on the ITK framework [18]. The extracted features were three-dimensional (3D) shape features, which describe the geometric shape of the segmented area and the geometric properties [15, 19, 20], intensity, histogram-based features which reduce the 3D information of a volume into a single histogram, and 3D texture-based features based on *grey level co-occurrence matrix* [17, 19, 20] or *grey level run length matrix*, also known as second-order statistics features, which are obtained calculating the relationship between adjacent voxels (Table 1) [20]. For texture-based 3D features, the mean and standard deviation of the values calculated along all the 3D directions were computed. Features extracted separately from the five images, distinguished using the code T0, T1, T2, T3, and T4, provide a description of the dynamic evolution of features over time due to the contrast wash-in/wash-out. Semeion’s training with input selection and testing (TWIST) algorithm [21] is based on an evolutionary strategy aimed at solving the features selection and training/test splitting problems simultaneously. To speed up the selection process, performances of selected features were evaluated by means of *k*-nearest neighbour (kNN), a fast and robust classification algorithm. The optimal feature set was used to build and validate the final kNN model.

**Table 1:** Features extracted from each image and time-point of the series

	Feature class			
	Intensity features^a^	Shape features^b^	GLCM features^c^ (mean and standard deviation computed along the 3D directions)	GLRLM features^d^ (mean and standard deviation computed along the 3D directions)
Features	Max	Eccentricity	Energy	Short run emphasis
	Min	Elongation	Entropy	Long run emphasis
	Mean	Major axis length (mm)	Inversion different moment	Grey level non-uniformity
	Sigma	Minor axis length (mm)	Inertia	Run length non-uniformity
	Variance	Volume (mm^3^)	Cluster shade	Low grey level run emphasis
	Integrated intensity		Cluster prominence	High grey level run emphasis
				Short run low grey level emphasis
				Short run high grey level emphasis
				Long run low grey level emphasis
				Long run high grey level emphasis

*3D* three-dimensional

^a^Intensity features: first order statistics calculated from pixel intensities

^b^Shape features: 3D shape descriptors

^c^Grey level co-occurrence matrix features: they are reported as average and standard deviation computed along all the three-dimensional directions

^d^Grey level run length matrix features: they are reported as average and standard deviation computed along all the 3D directions

### Statistical analysis

Performances of the optimal classification model were expressed in terms of sensitivity, specificity, accuracy, positive predictive value, negative predictive value, area under the curve (AUC) at receiver operating characteristic (ROC) analysis, positive likelihood ratio, and negative likelihood ratio. For each parameter, 95% confidence intervals (CI) were calculated according the binomial distribution. For *k*-nearest neighbour analysis, a *k* value of 3 was chosen. The probability of a case to belong to the positive class, *P* (+), was calculated on the basis of the class of the three nearest neighbours. *P* (+) can assume only three values: 1 if all three neighbours belongs to the positive class, 0.66 if 2 of the neighbours belongs to the positive class and one to the negative, 0.33 if 1 neighbour belongs to the positive class, and 0 if all 3 neighbours are of the negative class. The probability threshold applied to assign a case to the positive or negative class was set to 0.5. Probability values *P* (+) and class assignments were finally used to draw the ROC curve. The obtained ROC curve was used to obtain the AUC value.

## Results

### Population

A total of 1538 contrast-enhanced breast MRI examinations in female patients performed at our institution were retrieved from our database. After selection, following the inclusion criteria, a total of 68 lesions were analysed using the TWIST algorithm. Results are summarised in Table 2. The dataset obtained consisted of:

- Forty-five patients (median age 49 years, interquartile range [IQR] 44–54 years) had one enhancing focus each: 33 of them were benign with ≥ 1-year negative imaging follow-up, while 12 of them were malignant lesions. Among the 12 malignant lesions, for 8 lesions, the histopathology exams were retrieved (2 ductal cribriform, 5 ductal no special type, 1 ductal solid, and 1 ductal papillary) and were *in situ*; for the other 4 malignant lesions, no detailed pathologic information was available
- Eight patients (median age 46 years, IQR 44–56 years; median size 10 mm, IQR 7–14 mm) had one benign nodule each, confirmed after 5 years of MRI stability.
- Fifteen patients had one malignant lesions each (median age 55 years, IQR 45–66 years; median size 15 mm, IQR 10–24 mm) confirmed at the histopathology exam: 4 ductal cribriform, 7 ductal no special type, 2 ductal solid, 1 ductal papillary, and 1 tubular; of these 15 malignant lesions, 13 were invasive and 2 *in situ*.

**Table 2:** Study population (patients with enhancing foci and with unambiguous lesions)

Characteristic	Benign	Malignant	Total
Patients with enhancing foci	33	12	45
Patients with unambiguous lesions	8	15	23
Total	41	27	68
Patients aged 30–39 years	6	3	9
Patients aged 40–49 years	19	8	27
Patients aged 50–59 years	11	4	15
Patients aged 60–69 years	4	6	10
Patients aged 70–79 years	1	6	7
Total	41	27	68
Patients age (years)	47 (44–52)	58 (45–70)	52 (44–59)

Patients’ age is given as median (interquartile range)

### Feature extraction

The final features set obtained using the TWIST algorithm was reported in Table 3. In total, 43 features were extracted from five-time points, resulting in 215 features for each case, which described the dynamic evolution of the contrast agent in the focus. The most discriminating features extracted using the TWIST algorithm are also summarised in Table 3. Intensity- and texture-based features, which resulted to be the most important for the ML systems to differentiate benign from malignant lesions, were selected. As shown in Table 3, three of the extracted features refer to the pre-contrast image and 32 refer to images acquired after the contrast injection split over the different time points.

**Table 3:** Features extracted using the TWIST (training with input selection and testing) algorithm

Feature	Class	Statistics	Time point	Number of features
Energy	GLCM	SD	T0	3
Inversion different moment	GLCM	SD	T0	
Run length nonuniformity	GLRLM	SD	T0	
Entropy	GLCM	SD	T1	5
Long run emphasis	GLRLM	Mean	T1	
Inversion different moment	GLCM	SD	T1	
Cluster shade	GLCM	Mean	T1	
Long run high grey level emphasis	GLRLM	Mean	T1	
Entropy	GLCM	Mean	T2	12
Cluster shade	GLCM	Mean	T2	
Short run emphasis	GLRLM	SD	T2	
Short run low grey level emphasis	GLRLM	SD	T2	
Inertia	GLCM	SD	T2	
Cluster shade	GLCM	SD	T2	
Short run emphasis	GLRLM	SD	T2	
Long run emphasis	GLRLM	SD	T2	
Run length non-uniformity	GLRLM	Mean	T2	
Run length non-uniformity	GLRLM	SD	T2	
Short run low grey level emphasis	GLRLM	SD	T2	
Long run low grey level emphasis	GLRLM	Mean	T2	
Variance	Intensity		T3	8
Short run emphasis	GLRLM	SD	T3	
Run length non-uniformity	GLRLM	Mean	T3	
Low grey level run emphasis	GLRLM	Mean	T3	
Short run high grey level emphasis	GLRLM	Mean	T3	
Long run low grey level emphasis	GLRLM	SD	T3	
Max	Intensity		T3	
Inertia	GLCM	Mean	T3	
Integrated intensity	Intensity		T4	7
Cluster prominence	GLCM	SD	T4	
Grey level non-uniformity	GLRLM	Mean	T4	
Short run high grey level emphasis	GLRLM	SD	T4	
Long run low grey level emphasis	GLRLM	SD	T4	
Mean	Intensity		T4	
Long run emphasis	GLRLM	SD	T4	

T0, T1, T2, T3, and T4 represent the time-points of the dynamic series when the features were selected; the number represents the quantity of features selected for each time-point

*TWIST* Training with input selection and testing, *GLCM* Grey level co-occurrence matrix, *GLRLM* Grey level run length matrix, *SD* Standard deviation

The second result of the TWIST algorithm was the subdivision of the overall dataset into two statistically homogeneous features-based groups: group A and B consisted of 37 lesions (16 malignant and 21 benign) and of 31 lesions (11 malignant and 20 benign), respectively. On these, classification performances were calculated twice: firstly, using A as training set and B as test set and subsequently vice versa. Results of the final kNN model built with the 35 selected input variables are shown in Table 4. The classifier showed a sensitivity of 27/27 (100%, 95% CI 87–100%), a specificity of 37/41 (90%, 95% CI 77–97%), and an accuracy of 64/68 (94%, 95% CI 86–98%). In particular, 3 out of the 4 misclassified cases were enhancing foci and one was an unambiguous benign case. All errors were false positives.

**Table 4:** Diagnostic performance of the TWIST algorithm

	Training/testing sets			
	A/B	B/A	Total	95% confidence interval (%)
Sensitivity	100%	100%	100%	87–100
Specificity	90%	91%	90%	77–97
Accuracy	94%	95%	94%	86–98
Positive predictive value	85%	89%	87%	70–96
Negative predictive value	100%	100%	100%	91–100
True positives	11	16	27	
True negatives	18	19	37	
False positives	2	2	4	
False negatives	0	0	0	
Positive likelihood ratio	10	11	10	
Negative likelihood ratio	0	0	0	
Area under the curve	0.93	0.95		
	0.94*			

Results are presented for both analysis, in the second column for training set A and testing set B, in the third column with training set B and testing set A. In the fourth column, the total/mean value of the two results was calculated. Group A was composed of 37 cases, group B was composed of 31 cases.

*Area under the curve (AUC) average value between 0.93 (AUC A/B) and 0.95 (AUC B/A)

## Discussion

This preliminary study demonstrated that ML associated with radiomics may successfully distinguish malignant form benign enhancing foci on breast MRI examinations, potentially outperforming human assessment.

During this study after the patient selection step, the following steps were applied: an image registration, a manual lesion segmentation, and the feature extraction, selection, and classification step.

Feature selection and model validation are two significant methodological issues related to the application of ML, especially when dealing with small databases and a large number of variables. Feature selection is a procedure to identify and select the most informative variables to feed the statistical model. Validation is the evaluation step of the classification procedure, and its objective was to test if the procedure was generally applicable or fitted to the particular dataset used to build the classification system (overfitting). Validation can be carried out by splitting the dataset into two subsets, one used to train the classifier and one to test it. Training/testing sets splitting is critical especially when dealing with small datasets because random splitting can lead to statistically different sets containing not homogeneous information.

The proposed approach, ultimately based on a simple kNN classifier, provided 100% sensitivity and 90% specificity. Notably, all the misclassification errors were false positives that are preferred to false negatives from a clinical perspective. Features selected by the TWIST algorithm were mainly from contrast-enhanced images (eight features/image) while only three were selected from the unenhanced images. This suggests that contrast enhancement provides information that can be beneficially exploited by ML methods. Interestingly, the imaging time-point with the highest prediction relevance for the proposed ML system was the second (T2) after injection, with 12 features selected from this time-point, obtained 140 s after injection, taking into consideration our temporal resolution (60 s) and the initial 20 s of waiting time between the contrast agent injection and the first acquisition. This result was coherent, according to our breast radiologists, to what happens in the human-based diagnosis, where the first one-two subtracted series were the basis for diagnosis and usually represented on maximum intensity projections.

These preliminary results were evaluated in the general frame work of breast cancer management. GLOBOCAN [22] estimated 2,088,849 new breast cancer cases and 626,679 deaths worldwide in 2018. Only in the USA, 138,000 women die every year. In general, a woman has a 1 to 8 chance of developing breast cancer in her lifetime. High tumour stage at diagnosis was related to a worse prognosis for the patient and to higher costs for the health care systems [22, 23]. In fact, early breast cancer detection and prediction of response to treatments became the main objective of the actual clinical practice and research [24]. In recent years, breast MRI was included among the diagnostic methodologies as third level examination. Technical improvements, uprising availability of breast coils, and increasing care to minimise radiation has expanded the number of performed breast MRI investigations.

However, breast MRI can detect equivocal lesions, especially small enhancing foci, with imaging features that do not allow a clear human-based malignant/benign differentiation. The impact of the proposed ML method could be positive from the clinical, economic, and psychological point of view. Forecasting a likely benign enhancing focus would lead the patient to a more serene approach to the next follow-up. Conversely, defining an enhancing focus as probably malignant would suggest to carry out a targeted biopsy.

In this study, only data from the dynamic data set was used to build the statistical model. However, additional clinical data, not necessarily derived from imaging examinations, could be added to the dataset to enhance the performance and robustness of the method.

The small sample size used in this study was the main limitation to take into consideration. We are aware that with small samples and unbalanced dataset (*i.e.* datasets containing much more features than patients), the assessment of model reliability is weak and models are associated with a high risk of overfitting. In these cases, cross-validation methods could mitigate the risk of overfitting and provide more reliable estimation of models performances. Cross-validation methods were generally based on the random splitting of the available data in two subsets used for parameters estimation and testing respectively. TWIST, instead, adopts a statistically driven approach to split the available dataset into training and test sets that have been demonstrated to outperform traditional methods such as the k-fold approach and was successfully applied on several clinical datasets [25]. Another common problem with ML was imbalanced population samples, when cases are not equally distributed across classes. To avoid this problem, this study adopted a biased patient selection, with a high percentage of malignant patients included to balance benign cases. As a consequence, malignancy rate of the current study dataset was higher compared to other studies, for which a malignancy rate for foci from 2 to 23% [9–13] was reported.

Despite these limitations, this preliminary study suggests that ML could support the radiologist in the clinical decision making for enhancing foci on breast MRI. To turn this result into a robust clinical tool, two further steps should be carried out: first, the variability associated to differences in MRI sequences, devices and contrast agents should be addressed, and second, the interobserver variability in tumour segmentation as well as the patient-related variability must be investigated. The result of this work, if confirmed to a larger scale, might lead to decrease the uncertainty in the clinical decision making regarding enhancing foci on breast MRI.

## Data Availability

The datasets generated and/or analysed during the current study are available from the corresponding author on reasonable request.

## Abbreviations

3D: Three-dimensional
AUC: Area under the curve
BI-RADS: Breast Imaging Reporting and Data System
CI: Confidence interval
IQR: Interquartile range
kNN: *k*-nearest neighbour
ML: Machine learning
MRI: Magnetic resonance imaging
ROC: Receiver operating characteristic
TWIST: Training with input selection and testing
